# Addressing the relational determinants of health: Alison McCallum

**DOI:** 10.1093/eurpub/ckac129.477

**Published:** 2022-10-25

**Authors:** I Abdikarim

**Affiliations:** Public Health, Chaite UniversitatsMedizin, Berlin, Germany

## Abstract

**Problem:**

The persistence of systemic discrimination and inappropriate responses to unmet need by institutions in response to current challenges (COVID-19, austerity, war) requires a fresh look at the gaps in our response to inequities. We found evidence of avoidable inequity and harm associated with the relationships between individuals, communities, organizations, and governance structures. These have been described as the Relational Determinants of Health.

**Results:**

We undertook a scoping review to find research and policy interventions to address this problem. The relationships between individuals, communities and institutions that underpin how health is experienced and lives are lived were rarely considered explicitly in the design and implementation of interventions to address inequalities other than at local level or in response to individual trauma. We found academic work co-created with indigenous peoples did address trauma experienced due to historical structural violence and discrimination. It recognised the importance of land, culture, ceremony, and language in the organization of inter-personal, community and institutional relationships, codified the relational determinants of health and tested interventions that changed them. By applying assemblage theory, relationships with individuals and communities that had been previously excluded and harmed were being redesigned. These promising findings, replicated in different settings, indicate that relationships between communities and authorities that appear structural, are not fixed but can be primed to be changed for the better.

**Conclusions:**

To reduce the scale of future inequalities, it is essential that we chronicle and quantify the impact of the Relational Determinants of Health. We present a model that will enable methods for gathering evidence for change to be established, and to support interventions that will improve our research, advocacy, and policy responses to this challenge.

**Key messages:**

• Discrimination and unmet health need are associated with the quality of relationships between individuals, communities and institutions.

• Attention to the Relational Determinants of Health can help institutions reduce exclusion and harm by working with communities to redesign relationships.

